# *Phyllanthus emblica* Seed-Derived Hierarchically Porous Carbon Materials for High-Performance Supercapacitor Applications

**DOI:** 10.3390/ma15238335

**Published:** 2022-11-23

**Authors:** Lok Kumar Shrestha, Sabina Shahi, Chhabi Lal Gnawali, Mandira Pradhananga Adhikari, Rinita Rajbhandari, Bhadra P. Pokharel, Renzhi Ma, Rekha Goswami Shrestha, Katsuhiko Ariga

**Affiliations:** 1 International Center for Materials Nanoarchitectonics (WPI-MANA), National Institute for Materials Science (NIMS), 1-1 Namiki, Tsukuba 305-0044, Ibaraki, Japan; 2 Department of Materials Science, Faculty of Pure and Applied Sciences, University of Tsukuba, 1-1, Tennodai, Tsukuba 305-8573, Ibaraki, Japan; 3 Central Department of Chemistry, Tribhuvan University, Kirtipur, Kathmandu 44613, Nepal; 4 Department of Applied Sciences and Chemical Engineering, Pulchowk Campus, Institute of Engineering (IOE), Tribhuvan University, Lalitpur, Kathmandu 44700, Nepal; 5 Department of Advanced Materials Science, Graduate School of Frontier Sciences, The University of Tokyo, 5-1-5 Kashiwanoha, Chiba 277-8561, Kashiwa, Japan

**Keywords:** *Phyllanthus emblica* seed, chemical activation (KOH), hierarchically porous carbon, electrochemical energy storage, supercapacitor

## Abstract

The electrical double-layer supercapacitance performance of the nanoporous carbons prepared from the *Phyllanthus emblica* (Amala) seed by chemical activation using the potassium hydroxide (KOH) activator is reported. KOH activation was carried out at different temperatures (700–1000 °C) under nitrogen gas atmosphere, and in a three-electrode cell set-up the electrochemical measurements were performed in an aqueous 1 M sulfuric acid (H_2_SO_4_) solution. Because of the hierarchical pore structures with well-defined micro- and mesopores, *Phyllanthus emblica* seed-derived carbon materials exhibit high specific surface areas in the range of 1360 to 1946 m^2^ g^−1^, and the total pore volumes range from 0.664 to 1.328 cm^3^ g^−1^. The sample with the best surface area performed admirably as the supercapacitor electrode-material, achieving a high specific capacitance of 272 F g^−1^ at 1 A g^−1^. Furthermore, it sustained 60% capacitance at a high current density of 50 A g^−1^, followed by a remarkably long cycle-life of 98% after 10,000 subsequent charging/discharging cycles, demonstrating the electrode’s excellent rate-capability. These results show that the *Phyllanthus emblica* seed would have significant possibilities as a sustainable carbon-source for the preparing high-surface-area activated-carbons desired in high-energy-storage supercapacitors.

## 1. Introduction

Owing to the exceptionally high specific-surface-area, large porosity, well-defined pore-size distributions, interconnected mesopores, high electrical-conductivity and electrochemical stability, hierarchically micro- and mesopore architectures, and low cost, the nanoporous activated-carbons have received much interest as the efficient electrode in the high-performance supercapacitor, which is a leading electrochemical-energy-storage system [1,2,3,4,5,6,7,8,9,10,11,12]. In supercapacitors, also known as electrical double-layer capacitors (EDLCs), charges are stored by means of electrical double-layers at the surface of electrode material governed by the electrolyte-ion diffusion from the electrolyte solution to the electrode surface. Therefore, fast ion-transmission is the key to the high-power performance of supercapacitors. Due to the exceptional power (>400 kW kg^−1^), ultrarapid charging/discharging, exceptionally long cycle-life (>10,000), excellent rate capability, low internal resistance, easy operation, low-cost and nontoxicity, supercapacitors have been well explored in high-power-density electronic devices [13,14,15,16]. Nevertheless, since the energy density of supercapacitors is inferior and far lower, generally found in the range of 1–20 Wh kg^−1^, in comparison with commercial lithium-ion batteries (>160 Wh kg^−1^), supercapacitors are not yet commercially successful for daily utilities in high-energy storage and high-performance devices.

Extensive attempts have been made to optimize the energy performances of supercapacitors, mainly in two ways. The first is by optimizing electrode-materials structure and properties, to enhance the specific capacitance (*C*_s_), and the second is by expanding the potential operative frame of the supercapacitor cell (*V*), as the energy storage is proportional to *C*_s_*V*^2^. Generally, an aqueous electrolyte is replaced with non-aqueous organic electrolytes to expand the potential window. Similarly, several ionic liquids have also been explored, to enhance the energy performance of supercapacitors [17,18,19,20]. However, due to the safety issue and the need for sophisticated fabrication facilities, supercapacitors devices based on non-aqueous systems are less investigated. Meanwhile, materials chemists have put massive efforts into fabricating novel and sustainable electrode materials with excellent textural properties, to expand the specific capacitance of the supercapacitor electrodes.

Carbon-based materials, including fullerenes and fullerene-derived porous carbons, carbon nanotubes (CNTs) and carbon fibers, graphene oxide or reduced graphene oxides, porous carbon nanosheets, porous carbons obtained from the metal-organic frameworks (MOFs), metal carbides, carbon aerogels, and activated nanoporous carbons are the most broadly explored in the electrical double-layer capacitor [21,22,23,24,25,26,27,28,29,30]. Of these explored electrode materials, nanoporous-activated carbon materials are popular, as their production cost is low, they exhibit high thermochemical-stability, and offer a superior surface area with well-defined pores, which can accommodate sufficient amounts of electrolyte ions, enhancing energy-storage capacity. The specific surface area (ultrahigh) and well-developed porosity (large pore-volumes) are key parameters that enhance the specific capacitance of the electrode. Therefore, high conductivity nanoporous activated-carbons with hierarchical micro/mesopore structures have become encouraging materials in supercapacitors. The high microporosity of the electrode materials provides plenty of space in electrical double-layer formation, enhancing the charge storage (energy density), while high mesoporosity or a mesoporous channel-like structure accelerates ion transportation to the electrode surface, improving the rate performance and power density [31,32,33,34,35]. Nevertheless, there are several other important parameters that contribute to the overall supercapacitance performances of the electrode materials. These include the well-defined pore-size distributions, interconnectivity of the porous structure, pore framework-structure, wettability, shape and size of the carbon particle, and also heteroatom doping [36,37,38]. The proper optimizations of these parameters require the strategic design of the synthetic conditions for the synthesis of appropriate porous carbon materials from sustainable carbon sources for the application to high-energy-density advanced supercapacitor-applications.

Recently, nanoporous activated-carbons fabricated by the physical and chemical activation of biomass or biopolymers have been used in high-performance supercapacitor applications, due to their enormous specific surface areas, large porosity, excellent electrical conductivity, low cost with a simple fabrication process, and high chemical and thermal stability [39,40,41,42]. Self-heteroatom (nitrogen and oxygen) doping in biomass carbon additionally enhances the energy-storage capacity of supercapacitors by improving the wettability and pseudocapacitive behavior. Biomass is a plant-based material in nature, having lignocellulose as the key component. Pyrolytic decomposition of the biomass at a moderately lower temperature (200 to 300 °C) produces porous biochar, which, upon activation at higher temperatures (600–1100 °C) using different activators such as KOH, potassium carbonate (K_2_CO_3_), sodium hydroxide (NaOH), sulfuric acid (H_2_SO_4_), orthophosphoric acid (H_3_PO_4_), zinc chloride (ZnCl_2_), potassium chloride (KCl), calcium chloride (CaCl_2_), etc., results in high-surface-area hierarchically porous carbons [43,44,45,46,47,48]. Until now, different biomasses have been used as a carbon source to produce nanoporous activated-carbons, and their electrochemical-supercapacitance performance has been studied. It has been found that the surface textural-properties, and hence the energy-storage capacity of the biomass carbons, depend on the carbon sources themselves and other carbonization conditions, including the type of activator, carbonization temperature, impregnation ratio of the activator, heating ramp, and hold time [43,44,45,46,47,48,49,50]. For example, our group recently reported the supercapacitance-performance of carbon materials obtained by the ZnCl_2_ activation of *Nelumbo nucifera* (Lotus) seed powder from 600–1000 °C [51]. The carbon obtained by the carbonization at 800 °C displayed the highest surface area of 1316 m^2^ g^−1^, and the electrode achieved 272 F g^−1^ specific capacitance at 1 A g^−1^ with the superior cycle-life of 99% after 10,000 charging/discharging cycles. Using the KOH-activation process, the surface textural-properties of the Lotus seed carbon could be further enhanced. The ultrahigh surface area of 2489.6 m^2^ g^−1^ was achieved [52]. As a result, the supercapacitor electrode showed 379 F g^−1^ capacitance at 1 A g^−1^ with a good rate-performance sustaining 65% capacitance at 50 A g^−1^. The cycle performance was also exceptional. We have also found that *Choerospondias axillaris* (Lapsi) seed also has significant potential as the carbon source, as upon ZnCl_2_-activation it resulted in tremendously porous carbon with the specific surface area of 2272 m^2^ g^−1^. Owing to the huge surface-area and porosity with interconnected mesopores with a graphitic-framework structure, the electrode displayed 284 F g^−1^, a high specific capacitance, at 1 A g^−1^, followed by a long cycle-life of 99% after 10,000 cycles of successive charging and discharging [53]. In a separate report, Wu et al. [54] synthesized high-surface (2757 m^2^ g^−1^)- area hierarchically porous unique-microrods with self-nitrogen doping (1.34 wt%) from albizia flowers. The porous-microrod electrode attained exceptional capacitance of 406 F g^−1^ at a 0.5 A g^−1^, revealing the key role of nitrogen doping in energy-storage performance. Through the KOH activation, Sandhiya and coworkers [38] also successfully prepared self-nitrogen-doped porous carbons from poultry waste, and fabricated a flexible supercapacitor device that displayed a high energy-density of 23 Wh kg^−1^. Similarly, Chen and coworkers [55] prepared self-nitrogen-doped 3D carbons with porous structures, to make use of a waste carbon-source, cottonseed husk. The obtained carbon exhibited a honeycomb-like structure with interconnected hierarchical pores with a high surface area (1694 m^2^ g^−1^), and the prepared supercapacitor electrode achieved 238 F g^−1^, specific capacitance at 0.5 A g^−1^. Very recently, Zhang and coworkers [1,3] have successfully converted tobacco waste into porous carbon materials that exhibit high surface area by one-step carbonization-activation and ball milling, using the nano ZnO as template and activator [1], and similarly using hydrothermal pre-carbonization and KOH activation [3]. Carbon materials obtained from both methods achieved 220 F g^−1^ at 1 A g^−1^ (former) and 356 F g^−1^ at 0.5 A g^−1^ (later). These recent examples highlight the significant possibility for waste biomasses in producing high-performance supercapacitor electrode-materials.

In this contribution, the electrochemical energy-storage supercapacitance performance of the hierarchically porous carbon materials comprising both micro- and mesoporous structures obtained by the KOH activation of the novel carbon precursor, *Phyllanthus emblica* (Amala) seed, is reported. The structure, properties, and energy-storage performance of the Amala-seed carbon had not been previously studied. The high temperature (700 to 1000 °C) carbonizations of the KOH-impregnated biochar of *Phyllanthus emblica* (Amala) seed powder at a 1:1 weight ratio yielded porous amorphous carbons. Owing to the admirable surface area (1946 m^2^ g^−1^), good pore volume (1.115 cm^3^ g^−1^), hierarchical pore structures (micro- and mesopores), and interconnected pore architectures, the material displayed good performance as the electrode in the supercapacitor. The electrode displayed a specific capacitance of 272 F g^−1^ at 1 A g^−1^, complemented by 60.2% capacitance holding at 50 A g^−1^ and a remarkable cycle-life of 98.1% after 10,000 subsequent charging/discharging cycles, inferring the high-rate performance of the electrode. The textural properties and the electrochemical supercapacitance performance results indicate the tremendous potential of the *Phyllanthus emblica* seed, a bio-waste, as the sustainable carbon source for the mass production of porous carbon materials with hierarchical pore architectures at low cost, which is in high demand to improve the performance of supercapacitors.

## 2. Materials and Methods

### 2.1. KOH Activation of Phyllanthus emblica Seed Powder

The *Phyllanthus emblica* (Amala) seed was washed multiple times with hot Milli-Q filtered water and dried at 100 °C for 24 h before grinding into powder form. Biochar of Amala was obtained by heating the powder (500 g) at 300 °C for 6 h in air. The biochar (2 g) and KOH pellet (2 g) (1:1 weight ratio) were crushed using an agate mortar and pestle, transferred to the ceramic heating boat, and kept at 25 °C for 24 h before carbonizations at different temperatures (700, 800, 900, and 1000 °C). The carbonizations were performed in a tubular furnace (KOYO, Tokyo, Japan) in a constant flow of nitrogen gas (120 cc min^−1^) at the temperature ramp of 5 °C min^–1^, and a hold time of 3 h. Samples obtained after the carbonizations were treated with a dilute hydrochloric acid (HCl) solution (0.5 M:100 mL) to remove the excess KOH, and washed with Milli-Q filtered water several times (250 mL of water at a time), until the supernatant solution achieved pH 7. Finally, the materials were separated out by centrifugation and dried in a vacuum at 80 °C for 12 h. Prepared samples were further crushed in an agate mortar and referred to as AmC_K700, AmC_K800, AmC_K900, and AmC_K1000, corresponding to the carbonization temperature. The yield of the carbon material was found to be 0.50 g (25.3%:AmC_K700), 0.47 g (23.5%:AmC_K800), 0.44 g (22.1%:AmC_K900) and 0.40 g (20.1%:AmC_K1000). Amala seed powder (2 g) was also carbonized under the same conditions at 900 °C, without mixing with KOH for comparison as a control, and designated as AmP_900. The KOH-activation process for the creation of micro/mesoporous structures in the carbon skeleton is a complex phenomenon, and involves several processes. The generally accepted process during KOH activation involves the reduction of potassium compounds, oxidation of carbon, and other intermediate reactions:

6KOH + C → 2K + 2K_2_CO_3_ (1)

K_2_CO_3_ → K_2_O + CO_2_ (2)

CO_2_ + C → 2CO (3)

K_2_CO_3_ + 2C → 2K + 3CO (4)

K_2_O + C → 2K + CO (5)

### 2.2. Characterizations of Phyllanthus emblica Seed-Derived Carbon Materials

The carbon source, *Phyllanthus emblica* (Amala) seed powder, and the obtained KOH-activated porous carbons were characterized by Fourier-transform infrared (FTIR) (NICOLET iS20, Thermo Fisher Scientific, Waltham, MA, USA) spectroscopy, thermogravimetric analysis (TGA) (STA 2500, Regulus, NETZSCH, Wittelsbacherstraße, SELB, Germany), powder X-ray diffraction (XRD) (Rigaku X-ray diffractometer, RINT, Tokyo, Japan), Raman scattering (NRS-3100, JASCO, Tokyo, Japan), scanning electron microscopy (SEM: S-4800, Hitachi Co., Ltd., Tokyo, Japan) and transmission electron microscopy (TEM: JEOL, Akishima, Tokyo, Japan, Model JEM2100F operated at 200 kV). The resolution factor of the FTIR measurements was 1, with the total number of scans set to 32, and took 1 min 32 s for one sample measurement. The purity of the inert gas used in the TGA measurement was 99.99%. The TEM sample was prepared by drop-casting a dilute suspension of the carbon in ethanol on a carbon-coated copper grid, which was vacuum dried at 80 °C for 12 h. The surface textural-properties (surface area, pore volume, and pore-size distributions) were determined by analyzing the nitrogen sorption isotherms recorded on a Quantachrome Autosorb-iQ2, Boynton Beach, FL, USA.

### 2.3. Electrochemical Supercapacitance Performance Studies

The electrochemical supercapacitance of the Amala seed-derived porous carbon materials was studied using cyclic voltammetry (CV), galvanostatic charge/discharge (GCD), and electrochemical-impedance-spectroscopy (EIS) measurements in aqueous 1 M H_2_SO_4_ electrolyte solution in a three-electrode-cell set-up. First, the glassy-carbon electrode (GCE: outer and inner diameter of 10 and 5 mm, respectively), was modified as the working electrode. Carbon material (4 mg) was ground to fine powder and was added to a mixture of water and ethanol (2 mL:4:1 *v*/*v* ratio) and sonicated for 60 min to obtain a dispersion of carbon (2 mg mL^−1^). An aliquot of the suspension (3 µL) was cast on a clean and dry GCE, and dried at 60 °C for 2 h to solvent evaporation. The mass of the active electrode-material for each system was 6 × 10^−3^ mg. As a binder, a Nafion solution (5 µL: 5% in ethanol) was used. After the addition of the Nafion solution, the working electrode was dried at 80 °C for 2 h under vacuum. Using a platinum wire as the counter electrode, and Ag/AgCl as the reference electrode, the CV, GCD, and EIS measurements were performed on a CHI 660E workstation (CH Instruments, Inc. Austin, TX, USA). The specific capacitance (*C_s_*) of the electrode materials was calculated from the GCD curves obtained from the chronopotentiometry measurements as:

(6) $$C_{s}=\frac{I\times t_{d}}{m\times \Delta V}$$

where *I* (A), *t_d_* (s), *m* (g), and Δ*V* (V) correspond to the discharge current, the discharge time in the GCD plot, the mass of the active material on the GCE electrode, and the operating voltage (V), respectively.

## 3. Results

**Figure 1:**
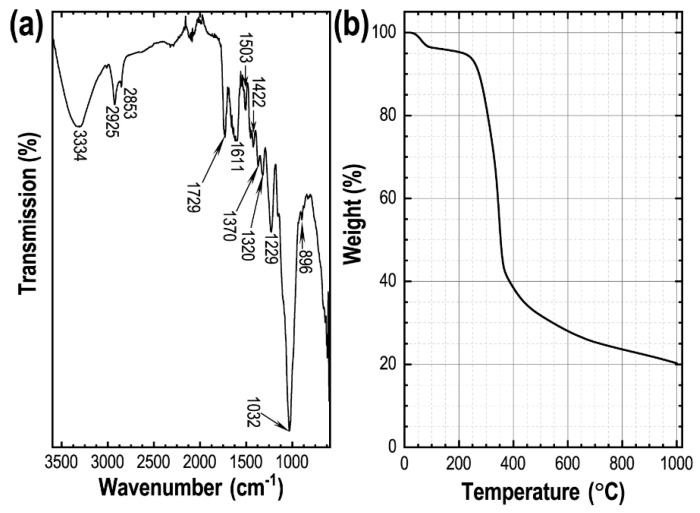
(**a**) FTIR spectrum of the precursor (*Phyllanthus emblica* seed powder); (**b**) the corresponding TGA curve.

The surface functionality of the biowaste, Amala seed powder, was studied by recording the FTIR spectrum. Oxygenated surface-functional-groups present in the precursor material can be seen in the FTIR spectrum (Figure 1a). A broad FTIR peak at 3334 cm^−1^ relates to the O–H (str.) of the moisture water and/or alcoholic group of the cellulose. At the same time, bands at 2925 and 2853 cm^−1^ indicate the aliphatic C–H (str.) of the cellulose. The band at 1729 cm^−1^ corresponds to the C=O (str.) of the unsaturated ester. A weak band around 1635 cm^−1^ suggests the moisture water in the precursor, and corresponds to the O–H (def.), and the band at 1611 cm^−1^ relates to the C=C (str.). Several FITR peaks were seen in the range of 1600–1000 cm^−1^, corresponding to C–H (def.) and C–O (str.), indicating cellulose and lignin, the major components of the biomass, a lignocellulosic material [56]. The oxygen functional group decreases drastically, due to high-temperature carbonizations in the AmC_K800, AmC_K900, and AmC_K1000 samples (Figure S1), while, due to the low-temperature carbonization, the AmC_K700 sample still contains oxygen functional groups (see Figure S1). Using thermogravimetric analysis, the pyrolytic decomposition of the Amala-seed powder is studied. The TGA curve reveals that the carbonization of the Amala seed proceeds in different stages (Figure 1b). Initially, the vaporization of moisture or crystallized water takes place below 200 °C (~5% moisture water is estimated from the TGA curve). The second stage includes the polymerization of cellulose and hemicellulose in the temperature range of 200–500 °C, and also involves the degradation of carbohydrates. As a result, volatile compounds and gases are released from the material, triggering a significant weight loss (~70% mass loss is observed: Figure 1b). In the final stage, the carbonation process takes place above 500 °C, and there is no significant mass loss in the TGA curve, indicating that the suitable temperature for the carbonization of the Amala seed is ≥500 °C. Judging from the thermogravimetric stability-profile of the precursor, we have performed KOH-activation of Amala-seed biochar at 700, 800, 900, and 1000 °C.

**Figure 2:**
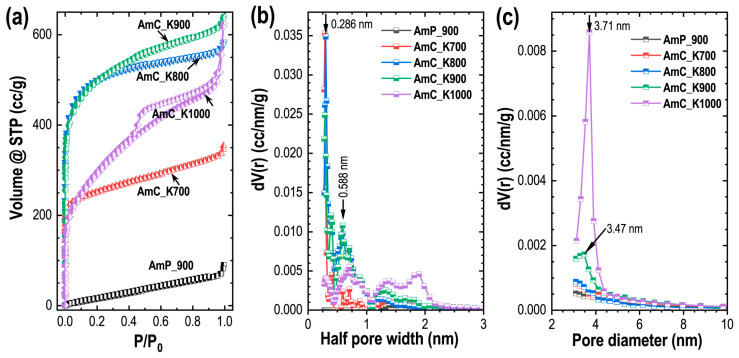
(**a**) Nitrogen sorption isotherms of AmP_900, AmC_K700, AmC_K800, AmC_K900 and AmC _K1000 samples; (**b**) pore-size-distribution curves obtained by the DFT analysis; and (**c**) pore-size-distribution curves obtained by the BJH analysis.

The porosity properties of the Amala-seed carbons were investigated by recording nitrogen sorption isotherms (Figure 2a), and the corresponding pore sizes and the histograms of their size distributions were obtained using the density-functional theory (DFT) method (Figure 2b) and the Barrett–Joyner–Halenda (BJH) model (Figure 2c). The DFT established an independent pore model, as proposed by Seaton and coworkers [57]. The model describes the adsorption isotherm as the collection of independent slit-shaped unconnected pores. The experimental data, i.e., the adsorption isotherm, N(*P*), which represents the average of all the pore sizes in the material can be expressed as:

(7) $$N\left(P\right)=\int_{H_{\min}}^{H_{\max}}\rho \left(P,H\right)f\left(H\right)\;dH$$

where *H*_min_ and *H*_max_, represent the smallest and largest pore widths, respectively, *ρ* (*P, H*) corresponds to the mean density of the nitrogen adsorbed in the pore width of *H* at pressure *P*, and *f*(*H*) represents the pore-size distributions. Detailed DFT calculation information can be found elsewhere [58,59].

As can be seen in the isotherm, the nitrogen adsorption in the directly carbonized sample (AmP_900 is low, indicating the lack of porosity. The isotherm follows Type-III behavior, which corresponds to nonporous materials, i.e., high-temperature carbonization of the Amala seed powder could not develop the large porosity. On the contrary, the nitrogen adsorption of the samples obtained by the KOH-activation is enormously large, and the nitrogen uptake is highly dependent on the carbonization temperature. All the carbon samples exhibit mixed Type-I/Type-IV sorption isotherms (Figure 2a), indicating the hierarchy in the pore structures composed of both the micro- and mesoporous structures. Substantial gas ingestion at a low relative pressure (P/P_0_ < 0.05: micropore filling) accompanied by steady adsorption at a high relative pressure (P/P_0_ > 0.1) with a clear hysteresis loop, validates the hierarchical bimodal-pore architectures, comprising micropores and mesopores. The presence of the hysteresis loop relates to the capillary condensation taking place in the mesopores [24,60,61]. At the low relative pressure, the nitrogen adsorption rises with the rise in the temperature of carbonization from 700 to 900 °C, suggesting the formation of more micropores. It is interesting to note that the nitrogen uptake decreases, and the hysteresis loop increases in the carbon sample carbonized at 1000 °C (AmC_K1000), due to the coalescence of the micropore resulting in the mesopore, due to the high temperature. The pore-size-distribution curves from the DFT (Figure 2b) show prominent peaks centered at 0.286 and 0.588 nm, confirming the micropores, while the pore-size-distribution curves obtained by the BJH analyses (Figure 2c) show distinct peaks at 3.47 nm (AmC_K900) and 3.71 nm (AmC_K1000), confirming the mesopores.

The surface textural-porosity properties of all the carbon samples are summarized in Table 1, which shows the key role of temperature in tuning the hierarchy in the pore structure of the KOH-activated Amala carbons, which is crucial in enhancing the electrolyte-ion transport and adsorption.

**Figure 3:**
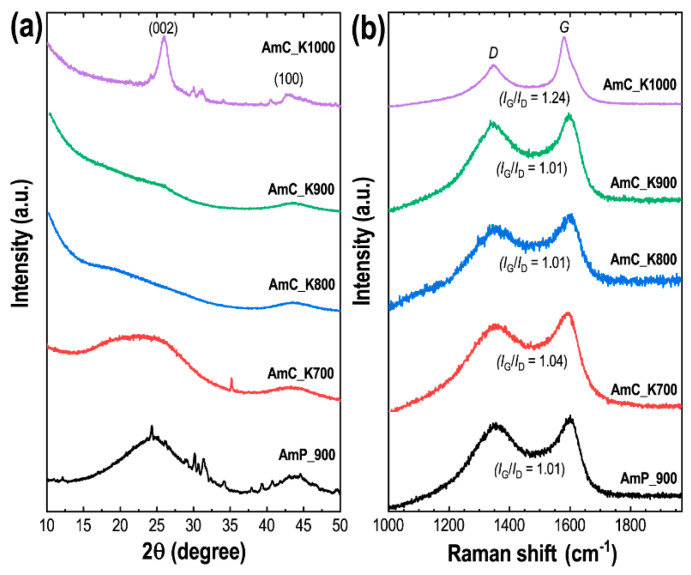
(**a**) pXRD patterns of AmP_900, AmC_K700, AmC_K800, AmC_K900, and AmC_K1000; (**b**) the corresponding Raman scattering spectra.

The structure of all the samples was studied using XRD and Raman scattering spectroscopy. XRD patterns mainly exhibit two wide diffraction bands at diffraction angles of 24 and 43° (Figure 3a), which relate to the (002) and (100) diffraction planes of the graphite-like structures of the disordered amorphous carbon. Such an amorphous structure has commonly been observed in the nanoporous activated-carbons derived from the natural precursors [56]. Since the directly carbonized sample AmP_900 was not washed with dilute HCl solution, it contains some impurities. For example, in the XRD patterns, in addition to (002) and (100), there exist other minor peaks corresponding to potassium hydrogen carbonate (Kalicinite). The intense XRD peak relating to the (002) plane in the AmC_K1000 sample is due to the formation of a highly graphitic carbon-structure, due to high-temperature carbonization. Raman scattering spectra of Amala carbons exhibit two prominent peaks (Figure 3b). The Raman band positioned at ~1348 cm^−1^ (*D* band) relates to disorder or the defective structure of carbon, and the Raman band at ~1595 cm^−1^ (*G* band) corresponds to the graphitic structure of the carbon, indicating the *E*_2g_ phonon of the *sp*^2^ carbon [62]. The extent of graphitization of the carbon material can be estimated by the intensity ratio of the *G* and *D* bands (*I_G_*/*I_D_*). The ratio of the samples carbonized up to 900 °C ca. 1.01, is close to that of the amorphous carbons prepared from other biomasses. However, the intensity ratio of the carbon sample carbonized at 1000 °C increases to 1.24, indicating higher graphitization, and thus improving the conductivity of the sample [63], which is advantageous for increasing the supercapacitance performance of the electrode.

**Figure 4:**
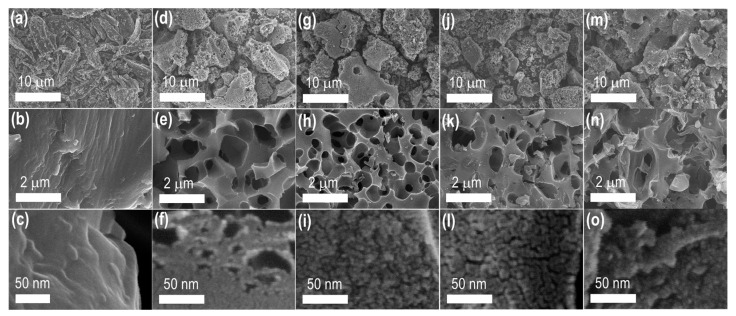
Surface morphology of the Amala seed carbons: SEM images of (**a**–**c**) AmP_900; (**d**–**f**) AmC_K700; (**g**–**i**) AmC_K800; (**j**–**l**) AmC_K900; and (**m**–**o**) AmC_K1000.

Using SEM imaging, the surface morphology of the Amala seed carbons was studied. SEM images reveal carbon particles with inhomogeneous shapes and sizes, with microporous channels as surface structures. Most carbon particles have a size ranging from a few to several tens of microns. The surface porosity of the AmP_900 (directly carbonized sample) is low (Figure 4a–c and Figure S2), which agrees with the nitrogen sorption data. In the high-resolution SEM image of AmP_900, one can hardly see the mesopore structure (Figure 4c). The surface porosity increases after the KOH activation (AmC_K700: Figure 4d–f and Figure S3, AmC_K800: Figure 4g–i and Figure S4, AmC_K900: Figure 4j–l and Figure S5, and AmC_K1000: Figure 4m–o and Figure S6). SEM images of the KOH-activated samples reveal plenty of macroporous channel-like structures, whose frameworks are composed of micro/mesopores, indicating the formation of hierarchical pore structures, and in agreement with the nitrogen-adsorption-isotherm data. Although micropores are not obvious, abundant mesopores are clearly seen in the KOH-activated samples (see high-resolution SEM images: Figure 4f,i,l,o). The mesopore sizes are rather polydisperse in the AmC_K700 (Figure 4f) and AmC_K1000 samples (Figure 4o). At the same time, the mesopores have a narrow size-distribution in the AmC_K800 (Figure 4i) and AmC_K900 (Figure 4l).

**Figure 5:**
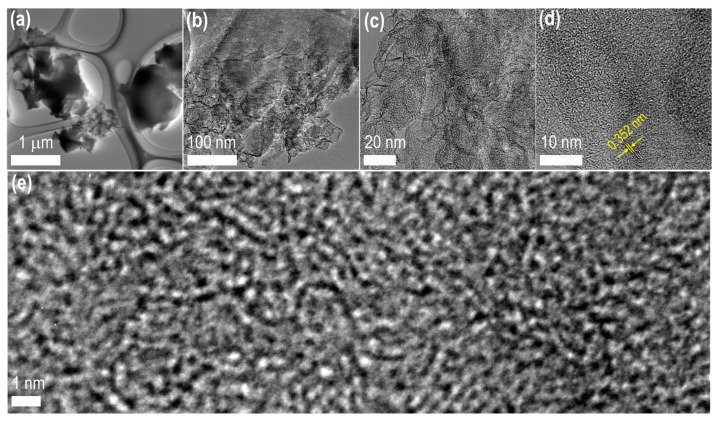
TEM observation of the optimal Amala-seed carbon sample, AmC_K900: (**a**–**c**) TEM images at different amplification; (**d**) high-resolution TEM image; (**e**) magnified HR-TEM image, showing microporous structure.

As the SEM observed, TEM images (Figure 5) show a well-developed micro/mesoporous structure. Carbon particles of a few microns in size, with a porous structure, can be witnessed in the low-resolution TEM image (Figure 5a). Due to thick carbon particles, only some of the pores are visible. The bright portion in this image refers to the porous structure. Magnified TEM images (Figure 5b,c) reveal abundant mesopores with graphitic frameworks, which enhance the electrolyte-ion transport to the micropores to form a high density of electrical double-layers. Micro/mesopore-architected carbon materials improve the supercapacitor electrodes’ rate performance [64]. The HR-TEM image (Figure 5d) further confirms the amorphous carbon structure with a partially graphitic system with a *d*-spacing of 0.352 nm, slightly higher than the bulk graphite (0.34 nm). Micropores with an interconnected framework can be realized in the magnified HR-TEM image (Figure 5e), demonstrating the material’s potential in high-energy storage supercapacitor-applications.

**Figure 6:**
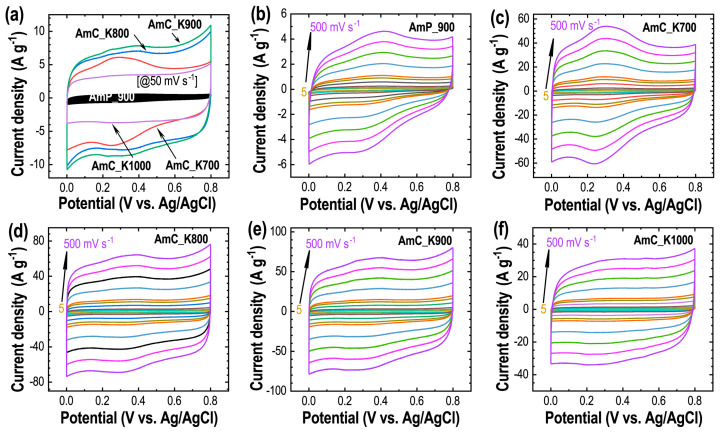
Electrochemical-energy-storage performance by CV measurements: (**a**) CV curves at fixed potential sweep of 50 mV s^−1^; and CV curves recorded at various sweep-rates (5, 10, 20, 50, 80, 100, 200, 300, 400, and 500 mV s^−1^) for (**b**) AmP_900; (**c**) AmC_K700; (**d**) AmC_K800; (**e**) AmC_K900; (**f**) AmC_K1000.

Motivated by the high surface area and hierarchically porous structures, supercapacitance performances of the Amala-seed carbons were investigated. Figure 6a compares the CV profiles of AmP_900, AmC_K700, AmC_K800, AmC_K900, and AmC_K1000, recorded at 50 mV s^−1^. All the profiles exhibit the typical behavior of the carbon electrode (EDLC-type energy-storage mechanism), quasi-rectangular CV profiles with a rapid current response upon exchange of the potential sweep. The CV curve of the AmC_K700 deviates from the ideal EDLC behavior, with weak redox peaks at 0.2–0.4 V, indicating the partial contribution of pseudocapacitance to the EDLC, due to the presence of oxygen functionalities in the carbon material, due to low-temperature carbonization [8]. The oxygen functionalities could be seen in the FTIR spectrum of the AmC_K700 (Figure S1). Note that the total integral current of the CV curve is extremely low in the AmP_900, due to the shortage of porosity. The current output is much higher in the KOH-activated sample, and the current increases with the carbonization temperature, achieving an upper limit in the sample carbonized at 900 °C (AmC_K900), and then decreases. This shows that AmC_K900 stored the highest energy among all the samples. This is well correlated with the surface area, as the greater the specific surface area, the higher the current output. Figure 6b–f shows the CV profiles of all the electrodes at various potential sweeps from 5–500 mV s^−1^, which show that the total-current output of each electrode escalates with the sweep rate, keeping the appearance of the CV curves unchanged from the semi-rectangular shape, even at 500 mV s^−1^, suggesting fast electrolyte-ion-transmission to the electrode surface via mesoporous structures, followed by excellent reversibility.

**Figure 7:**
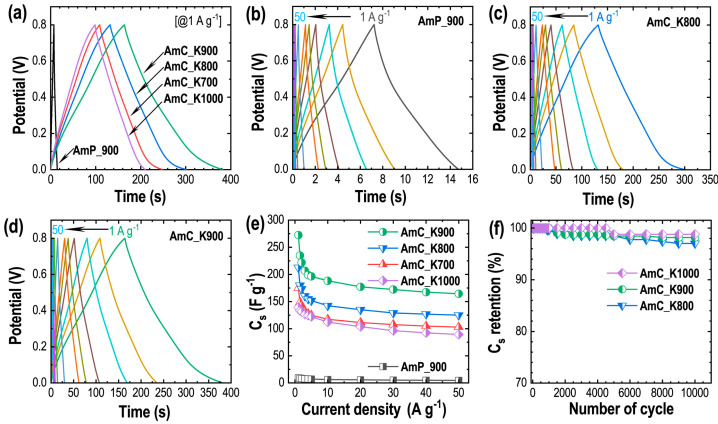
GCD measurement results: (**a**) Comparison of GCD profiles of all the electrodes recorded at 1 A g^−1^; GCD curves recorded at various current densities (1, 1.5, 2, 3, 4, 5, 10, 20, 30, 40, and 50 A g^−1^) for (**b**) AmP_900; (**c**) AmC_K700; (**d**) AmC_K800; (**e**) AmC_K900; (**f**) AmC_K1000.

The electrochemical-energy-storage properties studied by the GCD measurements is summarized (Figure 7). Figure 7a compares the GCD profiles of all the samples measured at 1 A g^−1^, a fixed current-density. Triangular-shaped symmetrical GCD profiles with a linear discharge again support the EDLC energy-storage-mechanism, with a well-balanced energy storage [64,65].

The discharge time in the GCD curve, which relates to the energy-storage capacity of the electrode material (Equation (6)), is longest in the AmC_K900, indicating the highest energy-storage capacity of this sample among all the samples studied [66,67]. This agrees with the surface-area properties of the materials (Table 1). As revealed in the CV results, the charge storage in the directly carbonized sample is low (shortest discharge time: Figure 7a). Similarly, due to the decrease in the specific surface area, the discharge time and hence the energy-storage capacity of the AmC_K1000 is lower, compared with the AmC_K700 and AmC_K800 samples (Table 1). Note that a slightly deviated triangular-shape of the GCD curves with a minor voltage fall is triggered, due to the oxygen functionality. Speedy electrolyte-ion transfer to the electrode surface, forming an electrical double-layer, is obvious in the GCD curves recorded at a higher current-density, up to 50 A g^−1^ (Figure 7b–d and Figure S7). The quasi-rectangular shape is retained, which infers balanced charge-storage and an excellent electrode-material rate performance, triggered by the hierarchically porous structures. Using Equation (6), the *C*_s_ were calculated. As expected, the directly carbonized sample showed only 9 F g^−1^, a very low specific capacitance, while the AmC_K900 electrode delivered a high *C*_s_ of 272 F g^−1^ (~30-fold enhancement in the capacitance, due to KOH activation). The *C*_s_ vs. current density plots for all the samples are shown in Figure 7e. The *C*_s_ of AmC_K900 is superior at all current densities from 1 to 50 A g^−1^, followed by AmC_K800 and AmC_K700. Although the *C*_s_ of these samples is higher, the rate performance of the AmC_K1000 is better, compared with other samples, due to the fast ion-diffusion, due to the high mesopore surface-area. The electrode retained 58% (AmC_K700), 58% (AmC_K800), 60% (AmC_K900), and 64% (AmC_K1000) of initial capacitance at a high current density of 50 A g^−1^ (Figure S8). The observed high-rate performance of the AmC_K1000 could also be due to the higher graphitization (Figure 3), which contributed to enhancing the conductivity. Note that a faster decrease in the calculated Cs with an increase in the current density from 1 to 5 A g^−1^ is caused by the poor ion-diffusion and low ion-penetration on the micropores of the electrode. The cycle performance recorded at 20 A g^−1^ (Figure 7f) for the selected samples shows excellent results, with a cycle life of more than 97% after the successive charging/discharging for 10,000 cycles, thus signifying the excellent potential of the Amala-seed carbon, as the electrode in the supercapacitor application with high-performance. Here again, the cycle performance of the AmC_K1000 sample is better than that of the AmC_K900 and AmC_K800, inferring the key role of the mesopore structure in the rate performance of the electrode. To sum up, the overall energy-storage performance of Amala carbon is good, and is superior to the carbons commercially available [68,69,70] and comparable to other similar carbon materials’ performances prepared from other precursors (Supplementary Materials Table S1).

**Figure 8:**
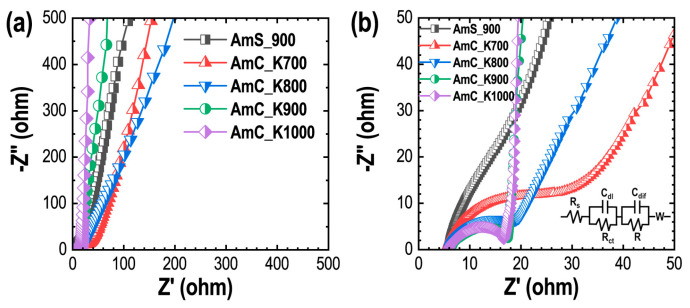
EIS Results: (**a**) Nyquist plots of Amala-seed carbon recorded in an aqueous 1 M H_2_SO_4_ solution from low to high frequencies (0.01 Hz to 100 kHz) at an amplitude of 5 mV; (**b**) the corresponding magnified plot. Inset of panel (**b**) representing equivalent circuit diagram, where *R*_s_, and *R*_ct_ represents solution or electrolyte resistance and charge-transfer resistance, respectively. Two capacitance and resistance combinations; *C*_dl_, *R*_ct_ refer to the double-layer formation and *C*_dif_, *R* represent the diffusion contribution. W represents the Warburg diffusion.

EIS measurements were conducted in the three-electrode cell set-up (frequency ranging from 0.01 Hz to 100 kHz), and the diffusion kinetics of ion transport at the electrode–electrolyte interface forming an electrical double-layer, was studied [71,72]. Nyquist plots of all the carbon samples, which plot the negative of the imaginary part vs. real components of the complex impedance, are shown in Figure 8a,b.

The plots of the KOH-activated samples consist of the semicircle at high frequency (Figure 8b) with nonvertical (AmC_K700 and AmC_K800) and vertical lines (AmC_K900 and AmC_K1000) in the low-frequency region. The semicircle represents the resistance of the system, and corresponds to the electrolyte resistance in the micropores of the electrode, the contact resistance, and the charge-transfer resistance. The low-frequency vertical lines signify the dominant capacitive behavior of the electrodes with faster ion-diffusion [73]. The equivalent series resistance (ESR), also known as the aggregate of the electrode resistance, electrolyte resistance, and contact resistance of the current collector and the electrode, was determined from the intersecting point on the real axis [73]. The calculated ESR values of all the samples: 5.8 Ω (AmP_900), 5.8 Ω (AmC_K700), 5.9 Ω (AmC_K800), 5.9 Ω (AmC_K900) and 5.8 Ω (AmC_K1000) are fairly low, and almost similar to each other, inferring that the energy-storage capacity of the electrodes is governed by surface-textural properties.

## 4. Conclusions

In conclusion, the KOH activation of the *Phyllanthus emblica* (Amala)-seed biochar resulted in the hierarchically porous activated-carbon materials possessing both micro- and mesopore structures of well-defined pore-size distributions. Because of the well-developed porosity, the amorphous carbons with a partially graphitic structure exhibited high specific surface area (1360 to 1946 m^2^ g^−1^) and large pore volumes, from 0.664 to 1.328 cm^3^ g^−1^, directly related to the temperature of carbonizations. Furthermore, all the carbon materials showed good supercapacitance properties in an aqueous electrolyte. The supercapacitor electrode made from a sample with the uppermost surface-area displayed excellent supercapacitive properties, achieving 272 F g^−1^ specific capacitance at 1 A g^−1^ with admirable rate-capability, retaining 60% capacitance at 50 A g^−1^, and a high current-density, followed by excellent cycle-life of 98% after the subsequent charging and discharging for 10,000 cycles. Note that the energy-storage capacity of the supercapacitor electrodes prepared from the porous carbons not only depends on the surface area, porosity, and pore size but also largely depends on the ionic/electrical conductivity hierarchy in the pore structure to enhance the ion transport, which could be improved by increasing the interconnectivity of the pores. Additionally, hetero-atom doping, including nitrogen and sulfur, advancing the graphitic nature of the framework structure, and boosting the wettability of the electrode increase the energy-storage capacity of the carbon-based supercapacitors. Functional porous-carbons with these features would be an asset to high-performance advanced supercapacitors. However, fabricating carbon materials with all these characteristic features is still challenging. Judging from their cost-effective production, rich textural-properties, and excellent electrochemical results, it may be concluded that the *Phyllanthus emblica* seed is a sustainable carbon-precursor (biowaste) for the mass production of porous activated-carbons with hierarchy in the pore architectures, which would be advantageous as the electrode materials in the high-performance electrical double-layer capacitor applications compared with other synthetic carbons. Furthermore, the process will be an asset to the green recycling of agricultural waste.

## Figures and Tables

**Table 1:** Surface textural properties of the directly carbonized sample, and the KOH-activated Amala seed carbons obtained by the carbonizations at different temperatures (700–1000 °C) ^1^.

Sample	*SSA* (m^2^ g^−1^)	*S*_micro_ (m^2^ g^−1^)	*S*_meso_ (m^2^ g^−1^)	*V*_p_ (cm^3^ g^−1^)	*V*_micro_ (cm^3^ g^−1^)	*W*_p_ (nm)	*D*_p_ (nm)
AmP_900	109	43	66	0.231	0.101	-	3.09
AmC_K700	1360	1268	92	0.664	0.491	0.286	3.09
AmC_K800	1779	1707	72	0.923	0.794	0.299	3.09
AmC_K900	1946	1799	147	1.115	0.885	0.286	3.47
AmC_K1000	1099	770	329	1.328	0.763	0.705	3.71

^1^*SSA* = total specific surface area, *S*_micro_ = micropore surface area, *S*_meso_ = mesopore surface area, *V*_p_ = pore volume (total), *V*_micro_ = pore volume from micropores, *W*_p_ = average half-pore width of the micropores, *D*_p_ = average pore diameter of the mesopores.

## Data Availability

The data presented in this study are available on request from the corresponding author.

## Supplementary Materials

The following supporting information can be downloaded at: https://www.mdpi.com/article/10.3390/ma15238335/s1, Figure S1: FTIR spectra of the carbonized samples; Figure S2: supplementary SEM image of AmP-900; Figure S3: extra SEM image of AmC-K700; Figure S4: supplementary SEM image of AmC-K800; Figure S5: supplementary SEM image of AmC-K900; Figure S6: supplementary SEM image of AmC-K1000; Figure S7: GCD curves of AmC_K700; Figure S8: Capacitance retention performance of AmC_K700, AmC_K800, AmC_K900, and AmC_K1000; Table S1: Comparison of the specific capacitance of Amala seed carbon with more activated carbons prepared from other biomasses [1,3,4,5,41,43,47,51,53,65,69,74,75,76,77,78,79,80,81,82,83,84,85,86,87,88].
