# Supplementary material for: Phyllanthus emblica Seed-Derived Hierarchically Porous Carbon Materials for High-Performance Supercapacitor Applications
Source: Materials (Basel). 2022 Nov 23;15(23):8335. doi: 10.3390/ma15238335 (PMC9739855; doi:10.3390/ma15238335)
Supplement: Supplementary file 1 [file materials-15-08335-s001.zip › materials-2034449-supplementary.pdf]

materials

Supplementary Information

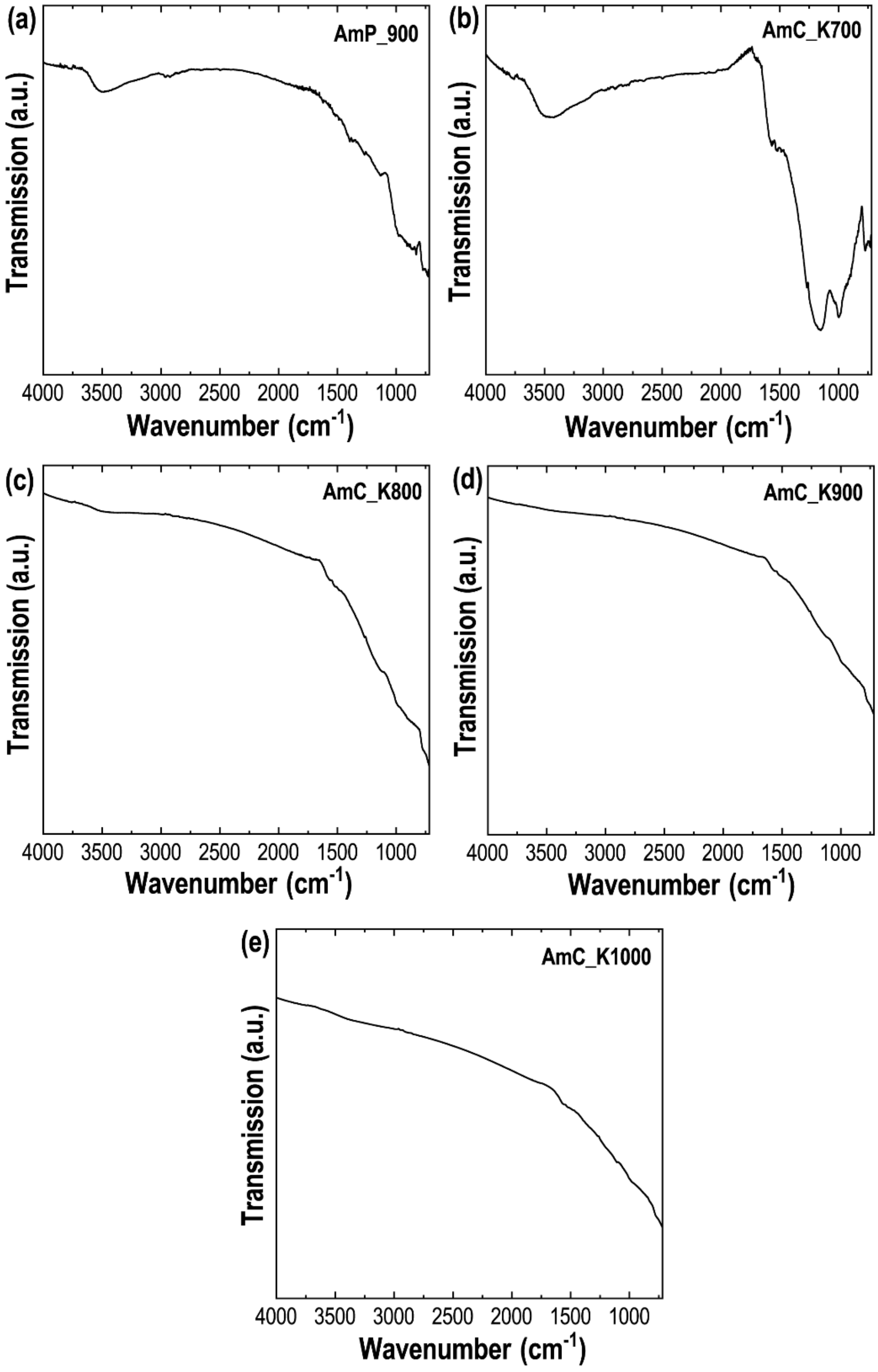

Figure S1. FTIR spectra of Amala seed derived porous carbon materials: (**a**) directly carbonized ample, AmP_900, and KOH activated carbons: (**b**) AmC_K700, (**c**) AmC_K800, (**d**) AmC_K900, and (**e**) AmC_K1000.

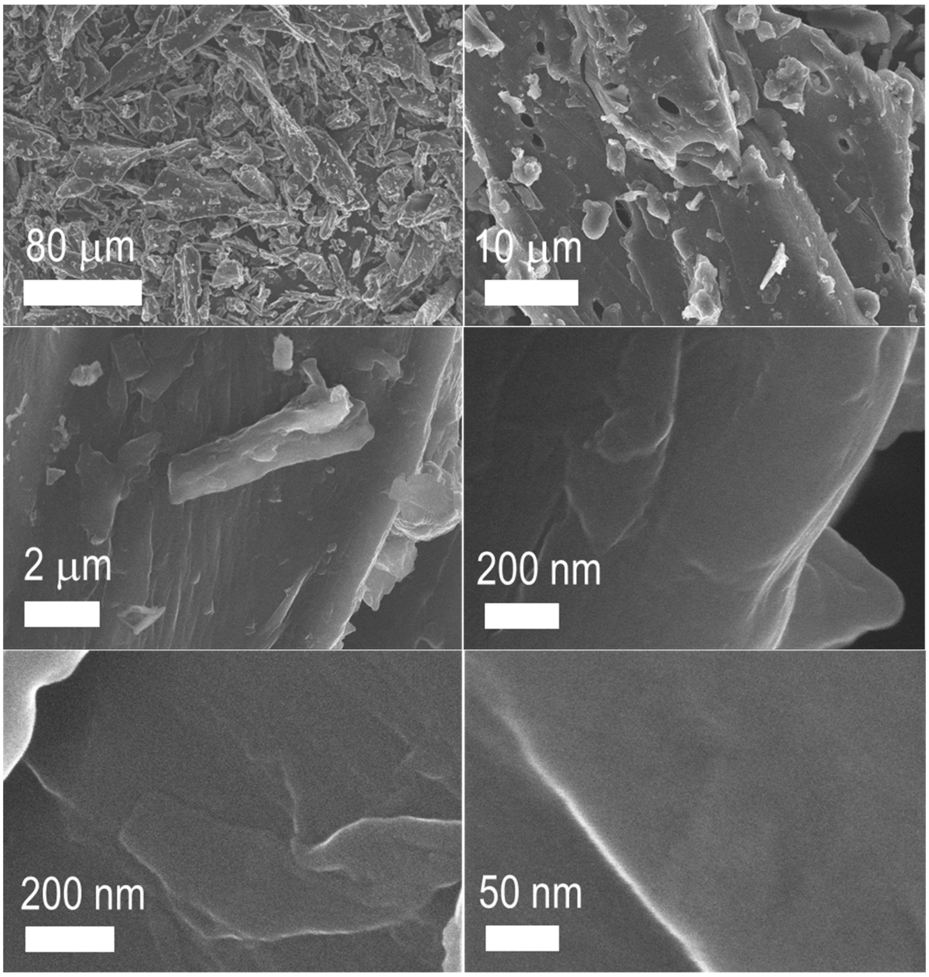

Figure S2. Additional SEM images of the directly carbonized Amala seed carbon (AmP_900).

.

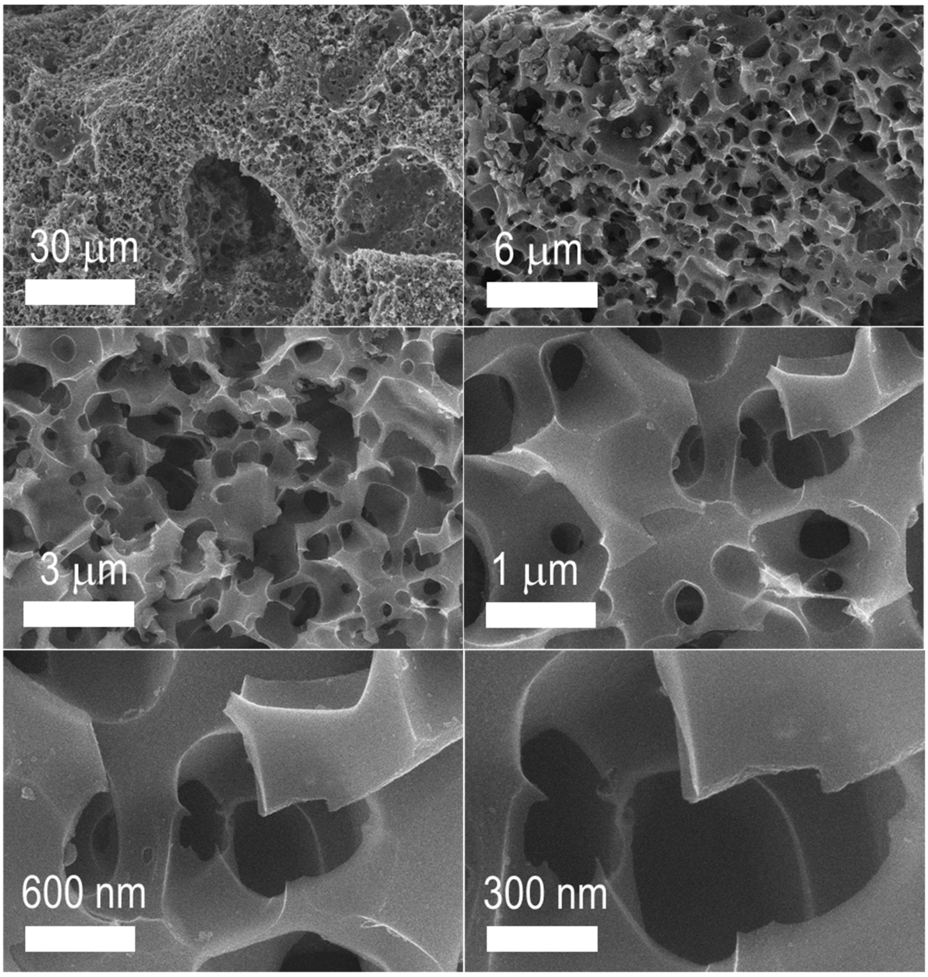

Figure S3. Additional SEM images of KOH-activated Amala seed carbon (AmC_K700).

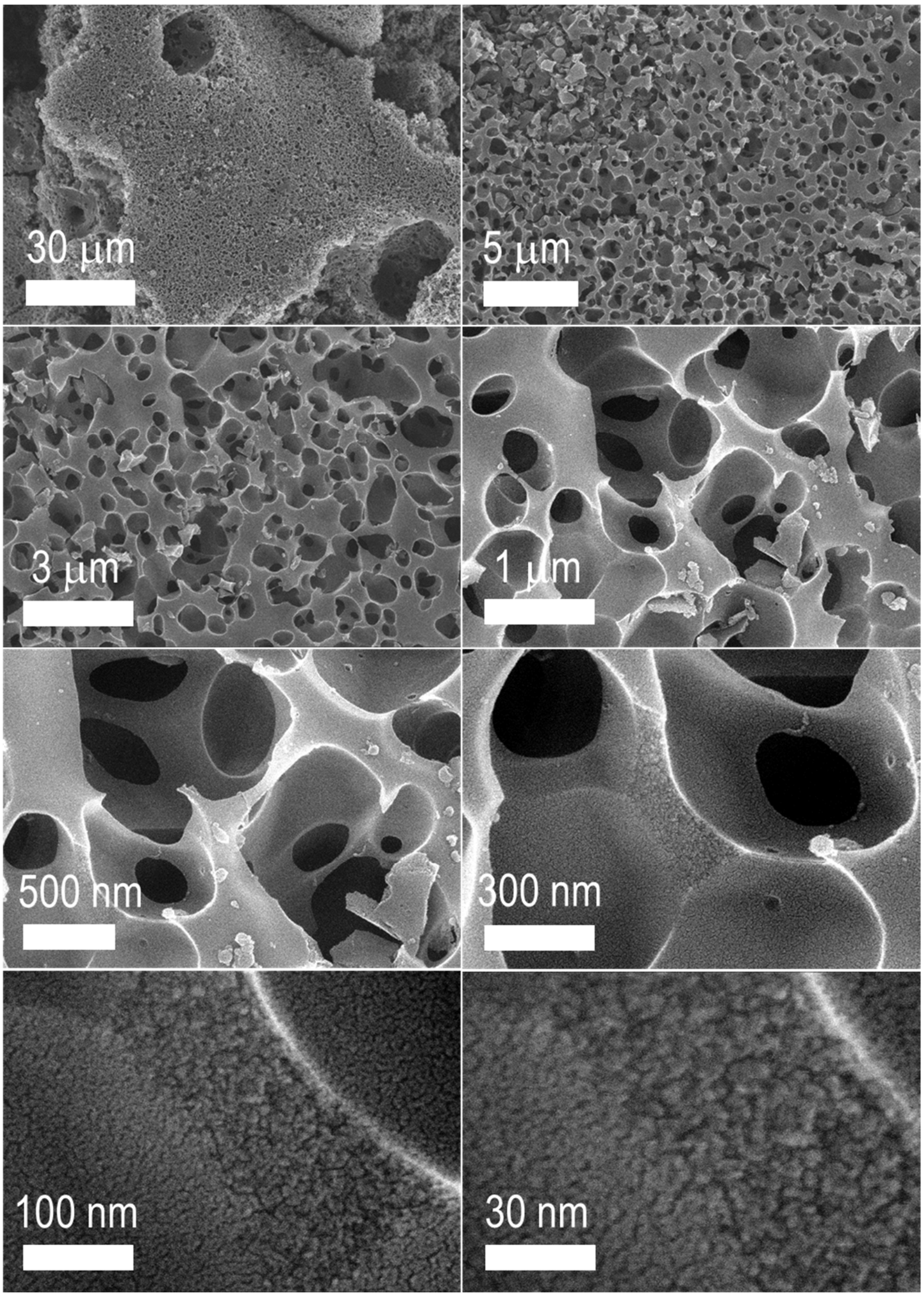

Figure S4. Additional SEM images of AmC_K800.

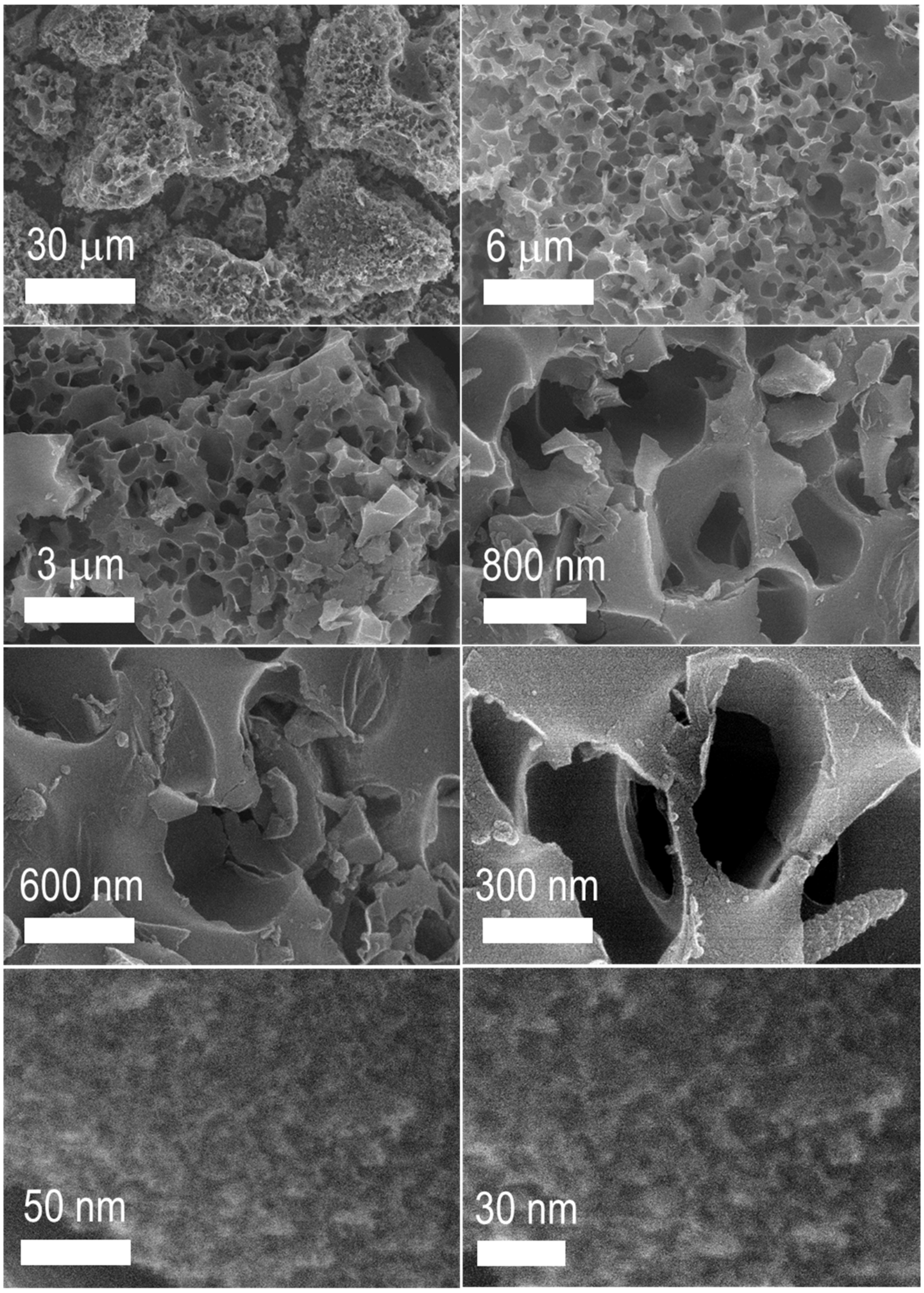

Figure S5. Additional SEM images of AmC_K900.

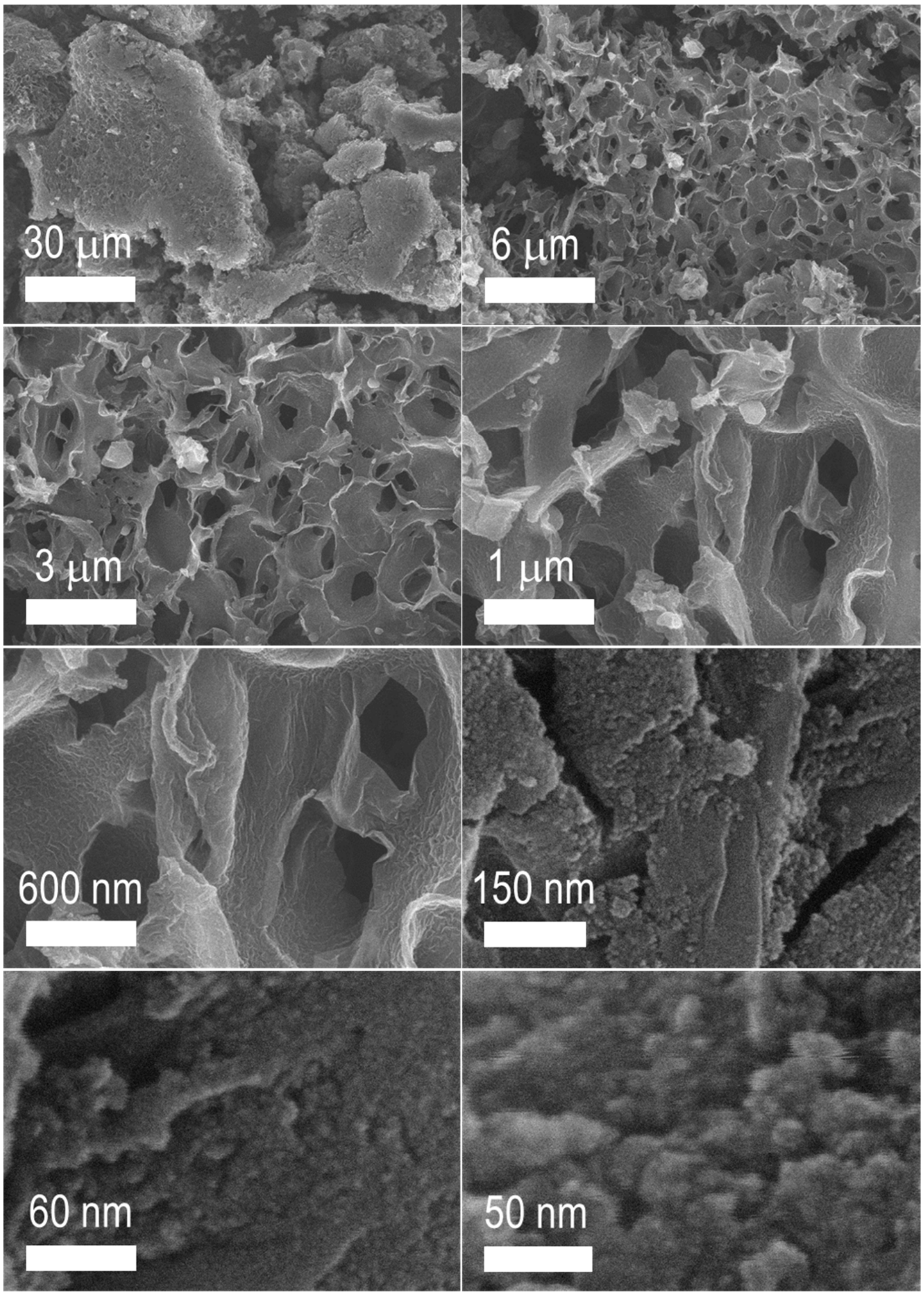

Figure S6. Additional SEM images of AmC_K1000.

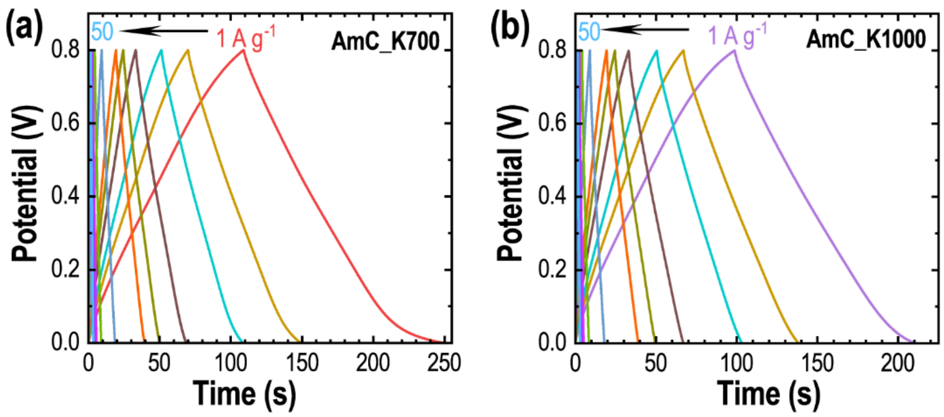

Figure S7. GCD curves recorded at different current density from 1 to 5 A g^{-1} for the electrode prepared using, (**a**) AmC_K700 sample; (**b**) AmC_K1000 sample.

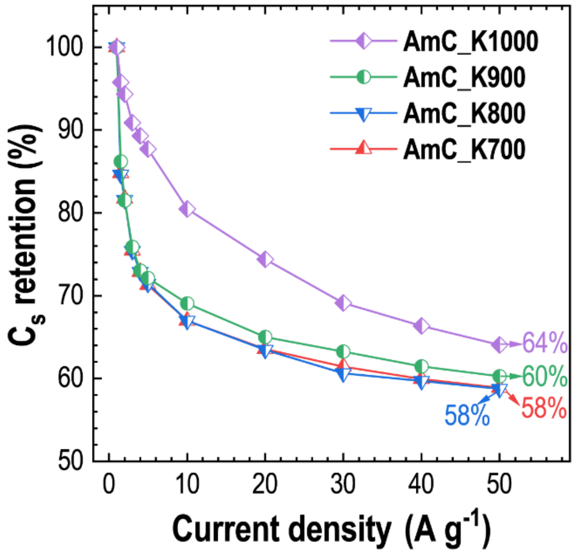

Figure S8. Capacitance retention performance of AmC_K700, AmC_K800, AmC_K900, and AmC_1000.

Table S1. The electrochemical supercapacitance performance of KOH-activated Amala seed carbon with similar other carbon materials derived from different biomasses.

Carbon Source	Electrolyte	Current Density (A g^{-1})	Specific Capacitance (F g^{-1})	Reference
Tobacco straw (ZnO templated)	6 M KOH	1	220	[1]
Tobacco waste (KOH-activated)	6 M KOH	0.5	356.5	[2]
Betelnut shells	1 M KOH	1	290	[3]
African maize Cob (H_2SO_4-activated)	6 M KOH	0.25	456	[4]
Date seed (KOH-activated)	1 M H_2SO_4	1	386	[5]
Lignocellulose carbon	1 M NaCl	1	172.9	[6]
Washnut seed (KOH-activated)	1 M H_2SO_4	1	288.7	[7]
Lotus seed ($ZnCl_2$-activated)	1 M H_2SO_4	1	272.9	[8]
Lapsi seed ($ZnCl_2$-activated)	1 M H_2SO_4	1	284.0	[9]
Aloe vera (KOH-activated)	1 M H_2SO_4	0.5	410	[10]
Bio-decomposited product (K_2CO_3-activated)	6 M KOH	0.05	209	[11]
Lotus leaf (KOH-activated)	6 M KOH	0.5	425	[12]
Washnut seed ($ZnCl_2$-activated)	1 M H_2SO_4	1	225.1	[13]
Jackfruit seed ($ZnCl_2$-activated)	1 M H_2SO_4	1	261.3	[14]
Pine Sawdust (CO_2-activated)	6 M KOH	1	225	[15]
Citrus bergamia peels (H_3PO_4 and $Mn(NO_3)_2$-activated)	6 M KOH	0.1	289	[16]
Prosopis juliflora wood (KOH-activated)	6 M KOH	0.5	588	[17]
Corncob (KOH-activated)	6 M KOH	0.5	382	[18]
Cotton fiber (NaOH-activated)	3 M KOH	0.3	222	[19]
Cottonseed hull (KOH-activated)	6 M KOH	0.5	304	[20]
Biomass-derived lignin	6 M KOH	0.5	348	[21]
Kraft lignin (CO_2-activated)	6 M KOH	0.1	155	[22]
salvia splendens (NaCl-activated)	6 M KOH	1	294	[23]
Quinoa (KOH-activated)	6 M KOH	1	330	[24]
Wood sawdust (KOH-activated)	6 M KOH	0.5	225	[25]
Wood	1 M H_2SO_4	0.5	260	[26]
Amala seed (KOH-Activated)	1 M H_2SO_4	1	272	This work

Reference

1. Jiang, B.; Cao, L.; Yuan, Q.; Ma, Z.; Huang, Z.; Lin, Z.; Zhang, P. Biomass Straw-Derived Porous Carbon Synthesized for Supercapacitor for Ball Milling. *Materials* **2022**, *15*, 924.
2. Huang, Z.; Qin, C.; Wang, J.; Cao, L.; Ma, Z.; Yuan, Q.; Lin, Z.; Zhang, P. Research on High-Value Utilization of Carbon Derived Tobacco Waste in Supercapacitors. *Materials* **2021**, *14*, 1714.
3. Ariharan, A.; Kim, S.-K. Three-Dimensional Hierarchical Porous Carbon Derived from Betelnut Shells for Supercapacitor Electrodes. *Materials* **2021**, *14*, 7793.
4. Kigozi, M.; Kali, R.; Bello, A.; Padya, B.; Kalu-Uka, G.M.; Wasswa, J.; Jain, P.K.; Onwualu, P.A.; Dzade, N.Y. Modified Activation Process for Supercapacitor Electrode Materials from African Maize Cob. *Materials* **2020**, *13*, 5412.
5. Shrestha, R.G.; Maji, S.; Mallick, A.K.; Jha, A.; Shrestha, R.M.; Rajbhandari, R.; Hill, J.P.; Ariga, K.; Shrestha, L.K. Hierarchically Porous Carbon from *Phoenix dactylifera* Seed for High-Performance Supercapacitor Applications. *Bull. Chem. Soc. Jpn.* **2022**, *95*, 1060-1067.

6. Lu, T.; Xu, X.; Zhang, S.; Pan, L.; Wang, Y.; Alshehri, S.M.; Ahamad, T.; Kim, M.; Na, J.; Hossain, Md.S.A.; Shapter, J.G.; Yamauchi, Y. High-Performance Capacitive Deionization by Lignocellulose-Derived Eco-Friendly Porous Carbon Materials. *Bull. Chem. Soc. Jpn.* **2020**, *93*, 1014-1019.
7. Shrestha, R.L.; Chaudhary, R.; Shrestha, R.G.; Shrestha, T.; Maji, S.; Ariga, K.; Shrestha, L.K.; Washnut Seed-Derived Ultrahigh Surface Area Nanoporous Carbons as High Rate Performance Electrode Material for Supercapacitors. *Bull. Chem. Soc. Jpn.* **2021**, *94*, 565-572.
8. Shrestha, R.L.; Chaudhary, R.; Shrestha, T.; Tamrakar, B.M.; Shrestha, R.G.; Maji, S.; Hill, J.P.; Ariga, K.; Shrestha, L.K. Nanoarchitectonics of Lotus Seed Derived Nanoporous Carbon Materials for Supercapacitor Applications. *Materials* **2020**, *13*, 5434.
9. Shrestha, L.K.; Shrestha, R.G.; Maji, S.; Pokharel, B.P.; Rajbhandari, R.; Shrestha, R.L.; Pradhananga, R.; Hill, J.P.; Ariga, K. High Surface Area Nanoporous Graphitic Carbon Materials Derived from Lapsi Seed with Enhanced Supercapacitance. *Nanomaterials* **2020**, *10*, 728.
10. Karnan, M.; Subramani, K.; Sudhan, N.; Ilayaraja, N.; Sathish, M. Aloe Vera Derived Activated High-Surface-Area Carbon for Flexible and High-Energy Supercapacitors. *ACS Appl. Mater. Interfaces* **2016**, *8*, 35191−35202.
11. Zhu, Y.; Chen, M.; Zhang, Y.; Zhao, W. A Biomass-Derived Nitrogen-Doped Porous Carbon for High-Energy Supercapacitor. *Carbon* **2018**, *140*, 404–412.
12. Liu, H.; Chen, W.; Zhang, R.; Ren, Y. Naturally O-N-S Co-Doped Carbon with Multiscale Pore Architecture Derived from Lotus Leaf Stem for High-Performance Supercapacitors. Bull. Chem. Soc. Jpn. 2021, 94, 1705─1714.
13. Shrestha, R.L.; Shrestha, T.; Tamrakar, B.M.; Shrestha, R.G.; Maji, S.; Ariga, K.; Shrestha, L.K. Nanoporous Carbon Materials Derived from Washnut Seed with Enhanced Supercapacitance. Materials 2020, 13, 2371.
14. Chaudhary, R.; Maji, S.; Shrestha, R.G.; Shrestha, R.L.; Shrestha, T.; Ariga, K.; Shrestha, L.K. Jackfruit Seed-Derived Na-noporous Carbon as the Electrode Material for Supercapacitors. C J. Carbon Res. 2020, 6, 73.
15. Gao, F.; Zhang, J.; Ren, M.; Ge, Y.; Chen, H.; Ma, X.; Hao, Q. Preparation and Characterization of Porous Carbons by Py-rolysis-CO2 Gasification of Pine Sawdust. Chem. Lett. 2020, 49, 652─655.
16. Gehrke, V.; Maron, G.K.; Rodriguez, L.D.S.; Alano, J.H.; Pereira, C.M.P.D.; Orlandi, M.O.; Carreño, N.L.V. Facile Preparation of a Novel Biomass-Derived H3PO4 and Mn(NO3)2 Activated Carbon from citrus bergamia Peels for High-Performance Su-percapacitors. Mater. Today Commun. 2021, 26, 101779.
17. Selvaraj, A.R.; Muthusamy, A.; Cho, I.; Kim, H.-J.; Senthil, K.; Prabakar, K. Ultrahigh Surface Area Biomass Derived 3D Hierarchical Porous Carbon Nanosheet Electrodes for High Energy Density Supercapacitors. Carbon 2021, 174, 463─474.
18. Song,Y.; Qu, W.W.; He, Y.H.; Yang, H.X.; Du, M.; Wang, A.J.; Yang, Q.; Chen, Y.Q. Synthesis and Processing Optimization of N-doped Hierarchical Porous Carbon Derived from Corncob for High Performance Supercapacitors. J. Energy Storage 2020, 32, 101877.
19. Liu, Y.; Shi, Z.; Gao, Y.; An, W.; Cao, Z.; Liu, J. Biomass-Swelling Assisted Synthesis of Hierarchical porous Carbon Fibers for Supercapacitor Electrodes. ACS Appl. Mater. Interfaces 2016, 8, 28283─28290.
20. Jiang, Y.; Zhang, Z.; Zhang, Y.; Zhou, X.; Wang, L.; Yasin, A.; Zhang, L. Bioresource Derived Porous Carbon from Cotton-seed Hull for Removal of Triclosan and Electrochemical Application. RSS Adv. 2018, 8, 42405─42414.
21. Cao, M.; Wang, Q.; Chang, W.; Huan, S.; Hu, Y.; Niu, Z.; Hang, G.; Cheng, H.; Wang, G. A Novel Strategy Combining Electrospraying and One-step Carbonization for the Preparation of Ultralight Honeycomb-like Multilayered Carbon from Biomass-derived Lignin. Carbon 2021, 179, 68─79.
22. Schlee, P.; Hosseinaei, O.; Baker, D.; Ladmér, A.; Tomani, P.; Mostazo-López, M. J.; Cazorla-Amorós, D.; Herou, S.; Titirici, M. -M. From Waste to Wealth: From Kraft Lignin to Free-standing Supercapacitors. Carbon 2019, 145, 470─480.
23. Liu, B.; Yang, M.; Chen, H.; Liu, Y.; Yang, D.; Li, H. Graphene-like Porous Carbon Nanosheets Derived from Salvia Splen-dens for High-rate Performance Supercapacitors. J. Power Sources 2018, 397, 1─10.
24. Sun, Y.; Xue, J.; Dong, S.; Zhang, Y.; An, Y.; Ding, B.; Zhang, T.; Dou, H.; Zhang, X. J. Biomass-derived Porous Carbon Electrodes for High-performance Supercapacitors. J. Mater. Sci. 2020, 55, 5166-5176.
25. Huang, Y.; Peng, L.; Liu, Y.; Zhao, G.; Chen, J. Y.; Yu, G. Biobased Nanoporous Active Carbon Fibers for High-performance Supercapacitors. ACS Appl. Mater. Interface 2016, 8, 15205─15215.
26. Chen, Z.; Zhou, H.; Hu, Y.; Lai, H.; Liu, L.; Zhong, L.; Peng, X. Wood-derived Lightweight and Elastic Carbon Aerogel for Pressure Sensing and Energy Storage. Adv. Funct. Mater. 2020, 30, 1910292.
